# Risk factors for BK virus infection in living-donor renal transplant recipients: a single-center study from China

**DOI:** 10.1080/0886022X.2018.1489843

**Published:** 2018-07-27

**Authors:** Ping Li, Dongrui Cheng, Jiqiu Wen, Kenan Xie, Xue Li, Xuefeng Ni, Shuming Ji, Jinsong Chen

**Affiliations:** National Clinical Research Center of Kidney Diseases, Jinling Hospital, Medical School of Nanjing University, Nanjing, China

**Keywords:** BK virus, renal transplantation, infection, risk factors, polymerase chain reaction

## Abstract

**Objectives:** BK virus (BKV) infection has become one of the main complications in renal transplant recipients (RTRs) with the arrival of newer potent immunosuppressive agents. However, reports on the epidemiology of BKV infection and risk factors in Chinese population after renal transplantation are scarce.

**Methods:** From June 2015 to July 2016, living-donor renal transplant recipients (LDRTRs) who routinely received the quantitative BKV DNA testing of urine and plasma samples using quantitative real-time polymerase chain reaction (PCR) for the first time after transplantation were selected, while dialysis patients and healthy living donors during that period served as controls. Potential variables were compared and analyzed using logistic regression model multivariate analysis to assess the BKV infection related factors in LDRTRs.

**Results:** Among the 52 LDRTRs identified, BKV DNA was detected in 16 urine samples (30.8%), significantly higher than that of dialysis patients (6.3%) and healthy living donors (4.2%) (*p* < .001). Nevertheless, no statistically significant difference wax noted between the latter two groups in urine samples (*p* = .842). Meanwhile, BKV DNA detection in blood samples was all negative in the three groups. Univariate analysis shown tacrolimus (Tac) trough level and lymphocyte percentage were associated with BKV infection in LDRTRs. Multivariate regression analysis also showed Tac trough level (HR, 1.644; *p* = .03), lymphocyte percentage (HR, 0.878; *p* = .026) were associated with BKV infection in LDRTRs.

**Conclusions:** In Chinese population, the incidence of BKV infection increased significantly after living-donor renal transplantation. Significantly increased Tac trough level and decreased lymphocyte percentage might be the risk factors for BKV infection in LDRTRs.

## Introduction

Human polyomavirus BK is a circular double-stranded DNA virus which was first reported in 1971 [1,2]. It is ubiquitous in healthy adults with a reported seroprevalence of >80% [3]. Following primary infection, BKV establishes latency in the uroepithelium and renal tubular epithelial cells where replication was controlled by the immune system. Nonetheless, in the setting of immunosuppression, such as pregnancy, acquired immune deficiency syndrome, and diabetes mellitus, the virus reactivates and replicates, triggering severe tissue or organ injury, the most prominent example of which is BK virus (BKV) associated-allograft nephropathy (BKVAN) [4,5].

With the introduction of new and potent immunosuppressive regimens, BKV infection rate increased gradually with BKVAN being a feared complication following kidney transplantation. In previous studies, 30%–50% of renal recipients developed BK viruria [6] and approximately one-third progressed to viremia [2], but only 1%–10% have BKVAN with graft loss in >50% of cases [4,7]. A variety of risk factors for BKV infection have been reported, which included patient characteristics, quality of the allograft and introduction of immunosuppressive regimens. The most consistently reported risk factor is immunosuppression with other risk factors being questioned.

The purpose of this study was to evaluate the incidence of BKV infection and associated risk factors in Chinese population after living renal transplantation.

## Materials and methods

### Patients

Between June 2015 and July 2016, 52 living renal allograft recipients who were routinely tested for quantitative BKV DNA in urine and plasma samples in Jinling Hospital were selected. In all the patients undergoing kidney transplantation, the panel reactive antibodies were all negative and CDC <10% preoperatively. Meanwhile, 167 dialysis patients and 71 healthy living donors were randomly chosen as control groups. We retrospectively collected clinical parameters such as immunosuppressive drug levels, white blood cell count, lymphocyte percentage, prior rejection episodes (T cell-mediated allograft rejection, antibody-mediated allograft rejection, or mixed rejection), and serum creatinine (Scr) level when BKV DNA positivity were ascertained. Demographics, human leukocyte antigen (HLA) mismatch and other clinical data of the three groups were extracted from Electronic Flow-up System.

All patients gave their informed consents to participate and voluntary received the urine/plasma BKV DNA detection.

### BKV DNA quantification in urine and plasma samples

BKV DNA quantifications were carried out by using the BKV nucleic acid quantitative detection kit (SinoMD, China), with ABI Prism 7500 Fast Renal Time PCR System (Applied Biosystems, USA). The lowest detection threshold of BKV DNA was 1 × 10^3^ copies/ml, and the linear detection range from 1 × 10^3^ to 5 × 10^8^ copies/ml. BKV DNA positivity was defined by detection of BKV DNA above the lower threshold in urine or plasma samples. According to the test results, the renal recipients were then divided into two groups, namely, group 1 composed of 16 BKV DNA positive patients while group 2 encompassed 36 BKV DNA negative patients.

### Immunosuppressive regimens

Post-transplant maintenance immunosuppressive therapy consisted of tacrolimus (Tac), mycophenolate mofetil (MMF), and prednisone (Pred).

Tac was started at 0.15 g/kg/d in two divided doses, targeting whole blood through levels of 6–10 ng/ml within six months. Progressive reduction of Tac was started from month +6, to reach target levels of 5–8 ng/ml through months +6 to 12, and 4–6 ng/ml thereafter. MMF was started at a dosage of 0.75 g twice daily. All patients received intravenous injection at a dose of 500 mg/d of methylprednisolone from intraoperative to postoperative days 2. Prednisone was started at 80 mg/d from postoperative days 3, reduced 10 mg daily to maintenance dosages of 20 mg/d, then gradually reduced to 10–15 mg/d at sixth month post-transplant, 5 mg/d at 12th month post-transplant and maintained constantly.

### Statistical analysis

Analysis was performed using SPSS software (version 18.0). Continuous variables were expressed as mean ± standard deviation (SD), or medians (range) for data with normal distribution or non-normal distribution, respectively, and were compared using the *t*-test. Categorical variables were shown as absolute frequencies, or percentage (%), Pearson Chi-square (or Fisher’s exact test) were used to analyze the data, as appropriate. Multivariate analysis of risk factors of BKV infection after renal transplantation was performed with logistic regression. *p* < .05 was considered significant.

## Results

### Characteristics of the study population

The causes for end-stage renal diseases for this cohort were summarized in Supplementary Table. As shown in Table 1, a total of 52 renal recipients with a mean age of 30.4 ± 8.6 years were enrolled in this study. Eighty-eight point five percent of recipients were male. The mean creatinine at the time of BKV positivity was 1.31 ± 0.25 mg/dl, and the duration from BKV positivity to renal transplantation was 0.5–50.5 months, among which 75% were within 6 months post-transplant, 12.5% were between 6 and 24 months post-transplant, and the rest were more than 24 months post-transplant.

**Table 1.** The clinical characteristic of population.

Characteristic	LDRTRs (*n* = 52)	Dialysis patients (*n* = 167)	Healthy living donors (*n* = 71)
Baseline demographics			
Age (years)	30.4 ± 8.6	34.0 ± 11.1	48.4 ± 7.7
Gender, *n* (%)			
Male, *n* (%)	46 (88.5)	122 (73.1)	23 (32.4)
Female, *n* (%)	6 (11.5)	45 (26.9)	48 (67.6)
Scr at detection (mg/dl)	1.31 ± 0.25	10.92 ± 4.20	0.83 ± 0.26
Quantitative BKV DNA load (×10^3^ copies/ml)			
Viruria, median (range)	4985 (2.2–500,000)	6.3 (2.4–1610)	23.1 (23–486)
viremia, median (range)	0	0	0
The interval from transplant to BKV DNA positive (months)	0.5–50.5	–	–

BKV: BK virus; LDRTRs: living-donor renal transplant recipients; Scr: serum creatinine.

Patients in the dialysis group were predominantly male (73.1%) with a mean age of 34.0 ± 11.1 years. Meanwhile, healthy living donors were mostly women (67.6%) with a mean age of 48.4 ± 7.7 years.

### Description of BKV infection of different groups

The results of quantitative BKV DNA detection showed that the rate of BKV DNA positivity in urine samples of renal recipients, dialysis patients, and healthy living donors were 16/52 (30.8%), 7/167 (6.3%), and 3/71 (4.2%), respectively, and the median quantitative BKV DNA load were 4985 (2.2–500,000), 6.3 (2.4–1610), 23.1 (23–486)×10^3^ copies/ml, respectively, whereas BKV DNA were all negative in plasma samples of the three groups, as shown in Table 1. Statistical analysis showed a statistically significant difference between renal recipients and the latter two groups on the rate of BKV positivity in urine samples, while no difference was noted among the latter two groups. Additionally, only the renal transplant patients received kidney biopsy during the research and none of them was diagnosed with BKVAN.

### Risk factors for BKV infection after living renal transplantation

Factors associated with BKV infection in renal transplant recipients (RTRs) by univariate analysis are shown in Table 2. In the BKV positive group, renal recipients had a higher Tac trough level than in the BKV negative group (8.09 ± 1.43 vs 6.79 ± 1.60, *p* = .008). Furthermore, renal recipients who had a lower lymphocyte percentage tended to have BKV infection. All other factors analyzed were not associated with BKV infection, including age, gender, number of HLA mismatch, induction therapy, MMF doses, prior rejection episodes, and white blood cell count.

**Table 2.** The related risk factors of BKV infection in living-donor renal transplantation recipients.

	Group 1 (*n* = 16)	Group 2 (*n* = 36)	*p*-value
Baseline demographics			
Age (years)	29.1 ± 7.2	31.0 ± 9.1	.472
Gender			
Male, *n* (%)	15 (93.7)	31 (86.1)	.653
Female, *n* (%)	1 (6.3)	5 (13.9)	
Creatinine at detection (mg/dl)	1.35 ± 0.29	1.29 ± 0.24	.408
Number of HLA mismatch			
HLA-I	1.81 ± 0.83	1.5 ± 0.97	.270
HLA-II	1.19 ± 1.11	1.47 ± 1.06	.381
Induction therapy			
Thymoglobulin, *n* (%)	4 (25.0)	10 (27.8)	1.000
Basiliximab, *n* (%)	12 (75.0)	26 (72.2)	
Immunosuppression regimens			
Tac mean trough level (ng/ml)	8.09 ± 1.43	6.79 ± 1.60	.008
MMF mean daily dose (g/d)	1.20 ± 0.23	1.17 ± 0.25	.624
Prior rejection episodes			
T cell-mediated allograft rejection, *n* (%)	0	1 (2.8)	.744
Antibody-mediated allograft rejection, *n* (%)	0	0	
Mixed rejection, *n* (%)	0	0	
Immunity index			
White blood cell mean count (×10^9^/l)	7.16 ± 2.45	7.56 ± 1.92	.53
Lymphocyte mean percentage (%)	22.52 ± 0.69	28.45 ± 7.08	.007

HLA: human leukocyte antigen; MMF: mycophenolate mofetil; Tac: tacrolimus.

Multivariate regression analysis also revealed Tac trough level (HR, 1.644; *p* = 0.03), lymphocyte percentage (HR, 0.878; *p* = 0.026) were associated with BKV infection in living renal allograft recipients, as presented in Table 3.

**Table 3.** Multivariate logistic regression analysis of BKV infection in living renal allograft recipients.

	HR [95%CI]	*p*-value
Immunosuppression		
Tac mean trough level	1.644 [1.051–2.574]	.030
Immunity index		
lymphocyte mean percentage	0.878 [0.783–0.984]	.026

Tac: tacrolimus.

## Discussion

With the introduction of more potent immunosuppressive regimens, BKV infection has emerged as a major complication in renal transplantation recipients, often leading to graft loss [2,4,8]. Transplant kidney biopsy has been the gold standard of diagnosing BKVAN [2]. However, studies have indicated that abundant BKVAN patients confirmed by biopsy often quickly manifested graft dysfunction, eventually leading to renal graft loss [9]. Therefore, efficient early diagnosis and treatment of BKV infection served to stabilize nephropathy, while noninvasive fluorescence quantitative PCR detection technology proved a valid tool to identify patients at risk of disease [10]. However, reports on the epidemiology of BKV infection and risk factors in Chinese population after living renal transplantation were limited.

This study retrospectively analyzed the testing results of BKV DNA in urine and plasma samples of living renal allograft recipients from June 2015 to July 2016 in Jingling Hospital. We found that the BKV-positivity rate in urine specimens of recipients was significantly higher than that of dialysis patients and healthy living donors, consistent with previous findings [3,11,12]. The BKV DNA detection was all negative in plasma samples of the three groups. In addition, for dialysis patients and healthy living donor, there was no difference in BKV infection both in urine or plasma samples, suggesting that dialysis did not increase the risk of BKV infection. Our finding also supported the notion that introduction of immunosuppressive regimens can increase the risk for BKV infection in living renal transplantation recipients.

The most consistent risk factors identified across studies for the BKV infection is the overall degree of immunosuppression [2,8]. Moreover, male gender, older recipients, HLA mismatch, low lymphocyte count were all considered to be associated with BKV in some studies [5,13,14]. Nevertheless, these findings have not been uniformly observed. In our study, all renal transplantation recipients received the immune inhibitors based on Tac, MMF, and Pred. Studies have suggested that Tac was the most permissive regimen for BKV infection [11]. Tac was shown to directly stimulate BKV replication via FKBP-12 binding in renal tubular epithelium [15]. We found that patients in the BKV positive group with a higher Tac trough level compared to the BKV negative group. It is speculated that high blood trough levels exposed to immunosuppression will stimulate BKV replication. Therefore, we considered that a higher Tac trough level also was a risk factor for BKV infection and advocated that regularly monitoring of BKV replication should be implemented in patients with a high Tac trough level, by which early diagnosis and treatment of BKV infection and improved graft function can be achieved. However, no association was found between the daily dose of MMF and BKV infection, although MMF has been shown to be a risk factor for BKV replication [16].

Additionally, we also found patients in the BKV positive group had a lower lymphocyte percentage compared to the BKV negative group, which is in line with previous studies [14]. This is of note because higher lymphocyte percentage is associated with enhanced immunity. Therefore, we should be more careful in terms of the decreased lymphocyte percentage in case of BKV replication in renal transplantation patients, especially for those who were not convenient to receive BKV detection.

Hirsch et al. [17] reported HLA mismatch was also a risk factor for BKV viremia. It was considered that antigen mismatching that lead to more episodes of acute rejection and tissue injury followed by cellular regeneration created condition favorable for BKV replication [18]. However, in our current analysis, there was no association between the BKV infection and the number as well as the types of HLA mismatch, partly because the effects of HLA on the evolution of BKV infection may be bidirectional and affected by other factors, such as the degree of immunosuppression, the incidence of rejection and other factors associated with immunity.

In conclusion, this study demonstrated the incidence of BKV infection in living-donor RTRs increased significantly, and there are significant correlations between BKV infection and Tac trough level, lymphocyte percentage in Chinese population. Thus, we advocate that the patients should be screened for BKV replication routinely pre-operatively and post-operatively, as well as the Tac trough level, and lymphocyte percentage so that appropriate immune suppression and improved long-term allograft survival can be expected.
